# Ligand-Directed Functional Selectivity at the Mu Opioid Receptor Revealed by Label-Free Integrative Pharmacology On-Target

**DOI:** 10.1371/journal.pone.0025643

**Published:** 2011-10-07

**Authors:** Megan Morse, Elizabeth Tran, Haiyan Sun, Robert Levenson, Ye Fang

**Affiliations:** 1 Department of Pharmacology, Pennsylvania State University College of Medicine, Hershey, Pennsylvania, United States of America; 2 Biochemical Technologies, Science and Technology Division, Corning Inc., Corning, New York, United States of America; The University of Kansas Medical Center, United States of America

## Abstract

Development of new opioid drugs that provide analgesia without producing dependence is important for pain treatment. Opioid agonist drugs exert their analgesia effects primarily by acting at the mu opioid receptor (MOR) sites. High-resolution differentiation of opioid ligands is crucial for the development of new lead drug candidates with better tolerance profiles. Here, we use a label-free integrative pharmacology on-target (iPOT) approach to characterize the functional selectivity of a library of known opioid ligands for the MOR. This approach is based on the ability to detect dynamic mass redistribution (DMR) arising from the activation of the MOR in living cells. DMR assays were performed in HEK-MOR cells with and without preconditioning with probe molecules using label-free resonant waveguide grating biosensors, wherein the probe molecules were used to modify the activity of specific signaling proteins downstream the MOR. DMR signals obtained were then translated into high resolution heat maps using similarity analysis based on a numerical matrix of DMR parameters. Our data indicate that the iPOT approach clearly differentiates functional selectivity for distinct MOR signaling pathways among different opioid ligands, thus opening new avenues to discover and quantify the functional selectivity of currently used and novel opioid receptor drugs.

## Introduction

Opioid receptors are a family of G protein-coupled receptors (GPCRs). This family consists of three principal receptor subtypes, termed mu (MOR), delta (DOR), and kappa (KOR) [1]. Opioid agonist drugs are potent analgesics that are used clinically for pain management [2]. Knockout mouse studies have shown that the MOR is the opioid receptor subtype primarily responsible for mediating the analgesic and rewarding effects of opioid agonist drugs [3]. However, chronic use of opioid agonist drugs may cause tolerance and dependence, thus limiting their therapeutic efficacy [3]. The progression of analgesic tolerance after the extended use of an opioid drug is believed to be linked to its unique ability to activate specific subset(s) of downstream signaling pathways of the MOR, a phenomenon termed functional selectivity [4]. Understanding the molecular mechanisms of opioid analgesia, tolerance and addiction is essential to the development of novel opioid drugs which can produce analgesia without leading to drug dependence. To achieve this goal, pharmacological assays that enable an integrated picture of the functional selectivity of opioid candidate drugs are required, so that lead compounds may be selected, prioritized and tested *in vivo*.

Currently, molecular assays that measure independent signaling parameters such as G-protein coupling or cAMP production dominate the drug discovery and development process [5]. Although these assays are useful in delineating specific aspects of GPCR biology and drug pharmacology, they are limited by two principal considerations. First, the plasticity of receptor on-off conformations [6]–[8], coupled with the oligomerization state of a particular GPCR [9], [10], makes it possible for many ligands to induce ligand-directed functional selectivity or biased agonism to receptor-mediated signaling [11]–[13]. However, measuring functional selectivity for a specific receptor agonist requires the application of multiple assays for screening and characterization. This process often produces contradictory results about the therapeutic potentials of drug candidates, complicating the drug screening process and making it difficult to select and prioritize lead compounds. Second, the ability to analyze receptor-mediated signaling using conventional pharmacological techniques is generally confined to a single readout parameter (*i.e.*, cAMP production or downstream phosphorylation events), rather than the integrated cellular response that occurs in response to ligand-mediated receptor activation [14], [15]. The inability of standard pharmacologic assays to measure integrated cellular responses in response to ligand-mediated receptor activation is reflected by the fact that there is generally a poor correlation between *in vitro* molecular assay results and the activity of drugs *in vivo* [16]. These considerations have made it difficult to assess the therapeutic potentials of active compounds using single node pharmacologic assays. New methodologies that enable an integrative pharmacological assessment of drug candidate molecules are needed.

To help overcome these difficulties, we have developed a high-resolution, label-free integrative pharmacology on-target (iPOT) [17] method to characterize the integrated response of cells to receptor activating ligands, and used this methodology to characterize a library of opioid receptor ligands. Key to this analysis is the dynamic mass redistribution (DMR) assay, which uses a label-free optical biosensor to non-invasively report ligand-induced responses in cells [18]. The resulting DMR signal is a reliable readout of GPCR functionality in various cell systems, wherein the dynamic redistribution of cellular contents is recorded in real-time with high sensitivity [19]. The DMR assay represents a powerful tool to delineate receptor signaling [19]–[22] and ligand pharmacology at the whole cell level [23]–[26]. DMR assay is also an effective method for screening novel pharmacologically active compounds [27].

In this study we have characterized a library of 42 opioid receptor ligands in HEK-293 cells stably expressing the MOR (HEK-MOR cells). By measuring DMR and cAMP production, we showed that at least 29 ligands in the library were agonists at MOR sites and activate distinct downstream signaling cascades. Our data indicate that the iPOT provides an integrated display of ligand-mediated receptor pharmacology and allows for a more effective prioritization of lead compounds for drug development.

## Results

### DMR characterization of mu opioid receptor

To characterize the MOR, we first performed DMR agonism assays. This assay monitors the DMR signals produced after stimulation with a ligand. We selected two endogenous opioid agonists (endomorphin-1 and endomorphin-2) and three exogenous opioid agonists (DAMGO, morphine and fentanyl) to gain a full perspective of agonist activity at the MOR utilizing DMR assay. Both morphine and fentanyl are clinically used opioid drugs. In addition, we characterized the DMR response of HEK-MOR cells utilizing known MOR selective (CTOP and β-funaltrexamine), as well as opioid non-selective (naloxone) antagonists. We performed a dose response analysis and determined the EC_50_ for each ligand tested and compared our results to the potency previously reported in the literature [28]–[30].

**Figure 1:**
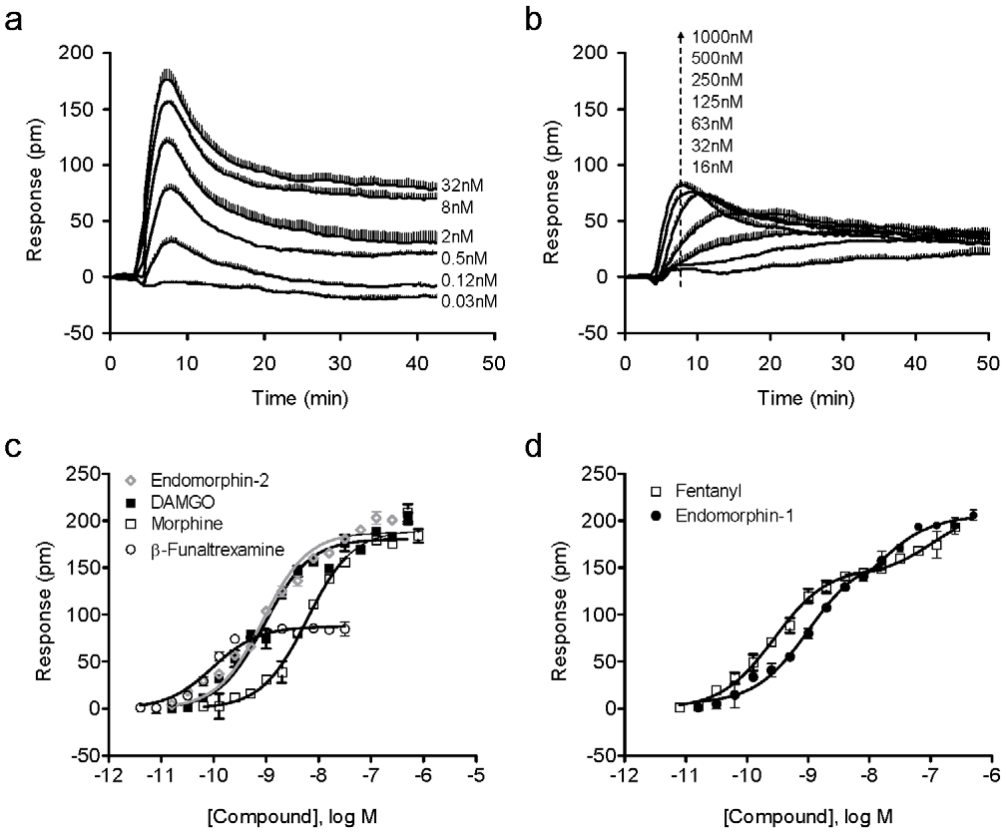
DMR characteristics of opioid ligands in HEK-MOR cells. (a) Real time kinetic responses of DAMGO at different doses; each graph represents the mean ± s.d. of 2 independent measurements (n = 6); (b) Real time kinetic responses of β-funaltreamine at different doses; each graph represents the mean ± s.d. of 2 independent measurements (n = 4); (c) The maximal DMR amplitudes as a function of ligand doses for endomorphin-2, DAMGO, morphine and β-funaltreamine; (d) The maximal DMR amplitudes as a function of ligand doses for fentanyl and endomorphin-1. All dose responses represent the mean ± s.d. of 2 independent measurements (n = 4), except for DAMGO (n = 6).

DMR assays showed that both DAMGO and β-funaltrexamine yielded a dose-dependent DMR response in HEK-MOR cells (Fig. 1a, and Fig. 1b, respectively). Similarly, endomorphin-1, endomorphin-2, morphine, and fentanyl all yielded dose-dependent DMR responses (Fig. 1c and Fig. 1d). β-Funaltrexamine, a selective MOR antagonist [31], led to a smaller maximum response than DAMGO (Fig. 1b), suggesting that this compound may be a partial agonist for the MOR. In contrast, both CTOP and naloxone led to a net-zero DMR in HEK-MOR cells (data not shown).

Non-linear regression analysis revealed that DAMGO, morphine, endomorphin-2, and β-funaltrexamine exhibited monophasic dose responses, all of which were best fit by a single-phase sigmoidal curve (Fig. 1c). This analysis revealed EC_50_ values of 0.93±0.12 nM (n = 6), 6.0±0.5 nM (n = 6), 0.86±0.07 nM (n = 4), and 0.10±0.02 nM (n = 4) for DAMGO, morphine, endomorphin-2, and β-funaltrexamine, respectively. However, both fentanyl and endomorphin-1 led to biphasic dose responses (Fig. 1d). Fentanyl's EC_50_ values were calculated to be 0.28±0.06 nM and 111±15 nM (n = 4), while endomorphin-1 produced EC_50_ values of 1.0±0.1 nM and 13.8±0.43 nM (n = 4). The EC_50_ values obtained using DMR assays were mostly consistent with their respective K_i_ values reported in literature (Table S1). The notable exception was fentanyl, whose EC_50_ was found to be 0.28 nM in DMR assays, ∼ 7x higher than the EC_50_ value (1.8 nM) reported previously [32].

**Figure 2:**
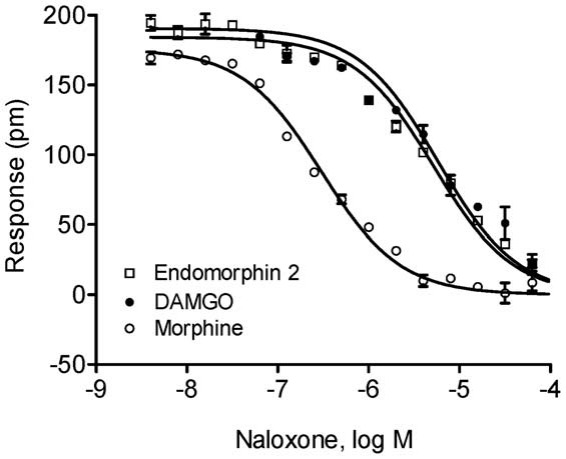
The blockage of MOR agonist-induced DMR by naloxone. The maximal amplitudes of the MOR agonist-induced DMR as a function of naloxone doses. All three agonists, endomorphin-2, DAMGO and morphine, were assayed at 64 nM. The data represents the mean ± s.d. of 2 independent measurements, each in duplicate (n = 4).

We next examined the ability of opioid antagonists to block the activity of MOR agonists using a two-step DMR antagonism assay, in which the cells were pretreated with an antagonist at different doses for about 1 hr, followed by the stimulation with an agonist for the MOR. Results showed that the DMR signals produced by endomorphin-2, DAMGO, and morphine were inhibited in a dose-dependent fashion by naloxone (Fig. 2). Naloxone is a well-characterized opioid antagonist with a high affinity for the MOR [33]. All three agonists were assayed at their respective EC_100_, 64 nM. However, naloxone most potently inhibited the morphine response with an IC_50_ value of 30.2±2.1 nM (n = 4). Naloxone exhibited IC_50_ values of 5.13±0.47 µM (n = 4), and 5.77±0.35 µM (n = 4) on endomorphin-2 and DAMGO, respectively. Taken together, the dose-dependent activation of the MOR by known MOR agonists, as well as the dose-dependent inhibition of these agonist responses by opioid antagonists, suggest that the real-time DMR assays accurately reflect the behavior of these ligands at MOR sites.

### Label-free on-target pharmacology profiling of opioid ligands

To examine the functional selectivity of opioid ligands, we used DMR assays to analyze the pharmacological properties of 42 opioid ligands in HEK-MOR cells. All ligands were profiled at 10 µM to achieve maximal signaling. The known potency of these ligands for the MOR is presented in supplemental material (Table S1). We designed a set of 13 assays to characterize MOR-mediated signaling produced by each of the ligands (Table 1). Beside the agonism profiles in parental HEK293 cells, all of the other assays were performed in HEK-MOR cells after pretreatment with a variety of probe molecules (Table 1). The choice of probe molecules was based on known signaling pathways downstream of the MOR activation. HEK-MOR cells pretreated with 0.1% DMSO were used as a control. These assays allowed us to discern receptor specificity, G-protein coupling, downstream kinase pathway selectivity, as well as any potential off-target effects.

**Table 1:** Assay protocols and DMR signals used for similarity analysis.

Assay #	Cell	Probe, pretreatment duration	DMR readout	Labels used in clustering
A	HEK293	none	Ligand, 10 µM	HEK-3, 9, 30
B	HEK-MOR	0.1% DMSO in buffer, 1 hr	Ligand, 10 µM	DMSO - Ligand-3, 9, 30
C	HEK-MOR	10 µM CTOP, 1 hr	Ligand, 10 µM	CTOP - Ligand-3, 9, 30
D	HEK-MOR	10 µM DAMGO, 1 hr	Ligand, 10 µM	DAMGO - Ligand-3, 9, 30
E	HEK-MOR	10 µM ligand, 1 hr	DAMGO, 10 µM	Ligand - DAMGO-3, 9, 30
F	HEK-MOR	10 µM ligand, 1 hr	CTOP, 10 µM	Ligand - CTOP-3, 9, 30
G	HEK-MOR	100ng/ml pertussis toxin, 20 hr	Ligand, 10 µM	PTx - Ligand-3, 9, 30
H	HEK-MOR	400ng/ml cholera toxin, 20 hr	Ligand, 10 µM	CTx - Ligand-3, 9, 30
I	HEK-MOR	10 µM forskolin, 1 hr	Ligand, 10 µM	FSK - Ligand-3, 9, 30
J	HEK-MOR	10 µM U0126, 1 hr	Ligand, 10 µM	U0126 - Ligand-3, 9, 30
K	HEK-MOR	10 µM SB202190, 1 hr	Ligand, 10 µM	SB202190 - Ligand-3, 9, 30
L	HEK-MOR	10 µM SP100625, 1 hr	Ligand, 10 µM	SP100625- Ligand-3, 9, 30
M	HEK-MOR	10 µM LY294002, 1 hr	Ligand, 10 µM	LY294002 - Ligand -3, 9, 30

**Figure 3:**
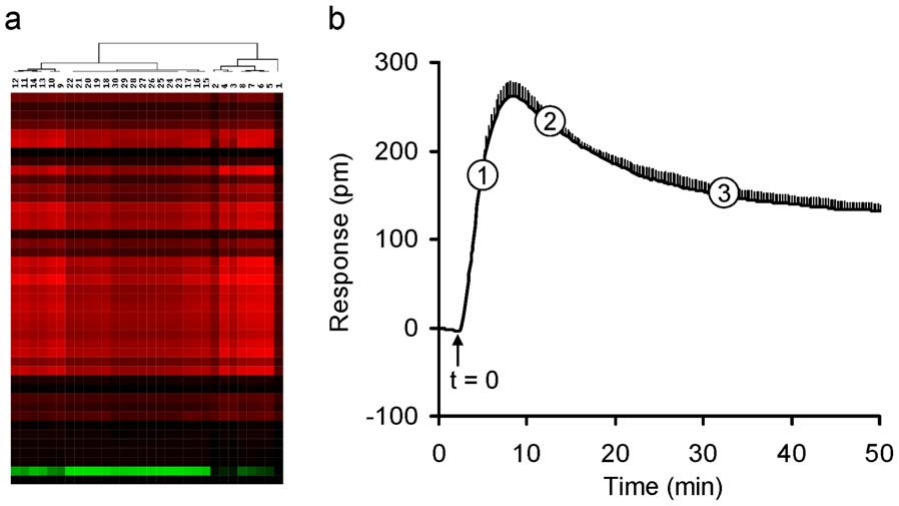
The numerical descriptor of opioid ligand pharmacology. (a) A false colored heat map of opioid ligand-induced DMR in the native HEK-MOR cells. The responses at distinct time points were subject to similarity analysis. Three major time domains were seen. (b) The DAMGO DMR signal in the native HEK-MOR cells and its three representative time point descriptors (3 min, 9 min and 30 min post-stimulation, as indicated by **1**, **2**, and **3**, respectively). The black arrow indicates the time (t = 0) when DAMGO was added. The graph represents the mean ± s.d. of 2 independent measurements, each in sixteen replicates (n = 32).

A DMR signal is recorded as a shift in resonant wavelength (picometer, pm), and is a real-time kinetic response with high temporal resolution (15 sec per data point) and long duration (∼ hours). The DMR signal can be considered to be a poly-dimensional coordinate at discrete time points, *i.e.*, a temporal series of DMR responses [17], [18], [34]. Therefore, DMR responses can be directly used as a basis for similarity analysis. Similarity analysis is a powerful means to determine the relationships (*i.e.*, similarity or distances) among different biological responses, particularly for large sets of biological data [35]. However, due to the large dimensions of DMR signals obtained under thirteen assay conditions for a specific ligand, a DMR numerical descriptor was developed for effective similarity analysis. We reduced the DMR dimensions to three distinct time points (3, 9, and 30 min post-stimulation) (Fig. 3). This dimensional reduction is based on clustering of time domains of the DMR response of all opioid ligands in DMSO-treated cells. Results showed that the ligand-induced DMR generally was propagated with three distinct time periods: short (1 to 8 minutes), intermediate (9 to 14 minutes), and late (15 to 50 minutes) (Fig. 3a). Similar patterns were obtained for all DMR signals under all thirteen conditions. Thus, we selected one time point from each period to represent each DMR (as indicated in Fig. 3b), and found that these time points adequately represent the key features of the ligand-induced responses. Thus, the thirteen DMR profiles of each ligand can be translated to a 39 dimensional coordinate.

**Figure 4:**
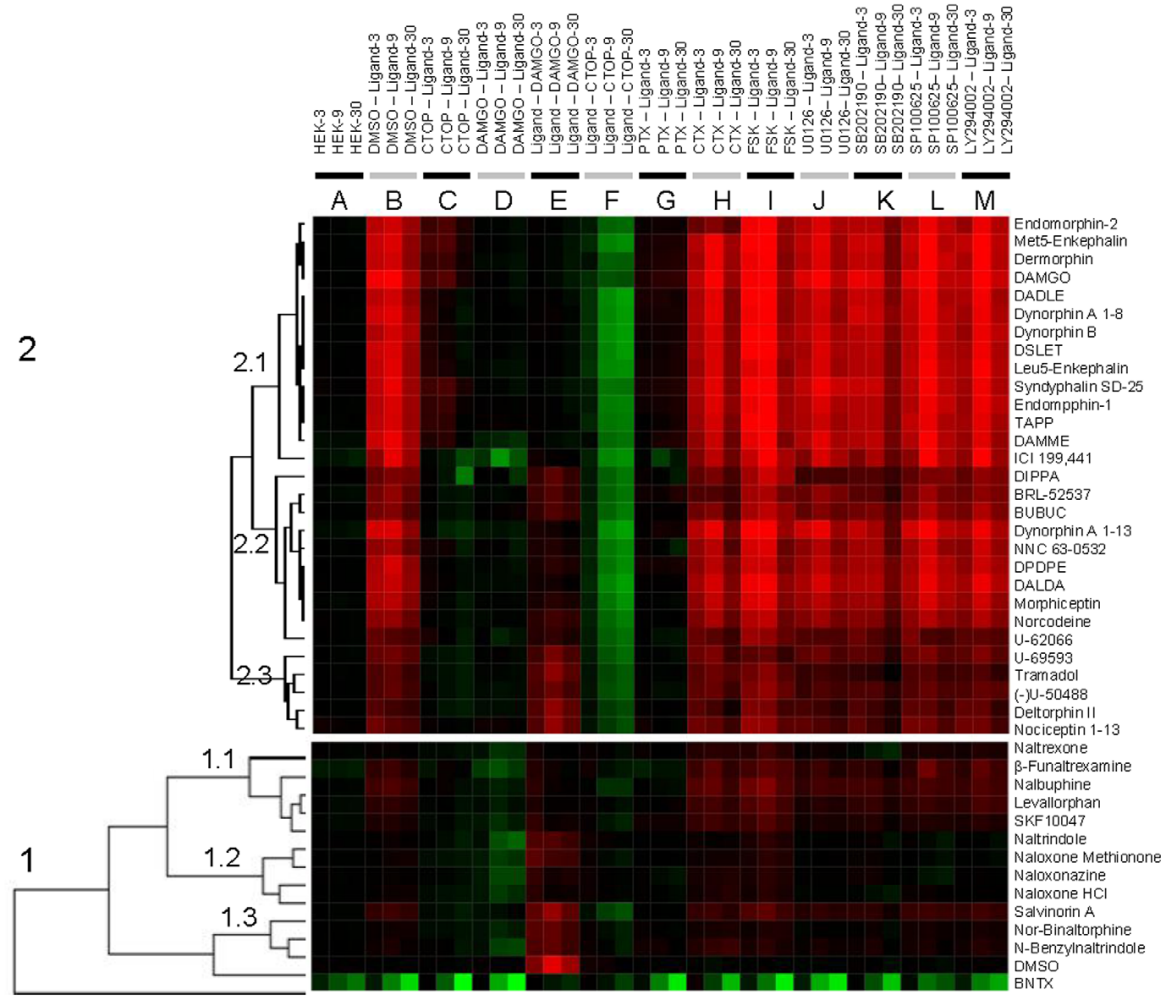
A false colored heat map of all 42 opioid ligands. The heat map was generated using similarity analysis of the DMR of all ligands under 13 assay conditions. 0.1% DMSO in the assay buffer was included as a control. In order to visualize the characteristics of a DMR for each assay, the three responses (3 min, 9 min, and 30 min) for each assay were grouped together. For each assay there are three adjacent columns to form a column group (A to M).

Application of an unsupervised Ward hierarchical clustering algorithm and Euclidean distance metrics led to a high resolution heat map of opioid ligands (Fig. 4). In order to visualize the characteristics of a DMR response for each assay, the three responses for each assay were grouped together (*i.e.*, for all ligands there are three adjacent columns, termed column group (A to M), for each assay in the heat map). Our data indicates that the opioid ligands can be grouped into two super clusters: antagonists (1) and agonists (2). Each super cluster can be further divided into several subclusters (1.1 to 1.3, 2.1 to 2.3 in Fig. 4). All ligands in the agonist cluster produced a positive DMR (P-DMR), in which at least one of the responses at the three time point is greater than 60 pm in the HEK-MOR cells. Conversely, all ligands in the antagonist cluster produced a DMR, in which all of the responses at the three time points are smaller than 60 pm in the HEK-MOR cells. We postulated that similarity analysis allows us to derive hypotheses regarding the mechanisms of action of the opioid ligands tested, and to explore the relationships between structure, function and pharmacological activity of the different opioid ligands.

### Receptor Specificity

To determine the specificity of opioid ligands for the MOR, we used four distinct two-step DMR assays: (1) Cells were pretreated with the MOR antagonist CTOP, followed by stimulation with an opioid ligand. The ability of CTOP to block the ligand-induced DMR indicates that the agonism profile of a ligand is due to the activation of the MOR. (2) Cells were pretreated with the MOR agonist DAMGO, followed by stimulation with an opioid ligand. The ability of the opioid ligand to alter the DAMGO response indicates that the ligand deactivates the DAMGO-activated cells. (3) Cells were pretreated with an opioid ligand, followed by stimulation with CTOP. The CTOP response indicates the MOR specific agonism of the ligands, since CTOP specifically reversed a MOR agonist-induced DMR. 4) Cells were pretreated with an opioid ligand, followed by stimulation with DAMGO, in which the DAMGO response indicated MOR specific agonism or antagonism of the ligand. Together with the agonism profiles in both parental HEK293 and HEK-MOR cells, these assays allowed us to determine the specificity, relative potency, efficacy, and signaling properties of each of the opioid ligands.

**Figure 5:**
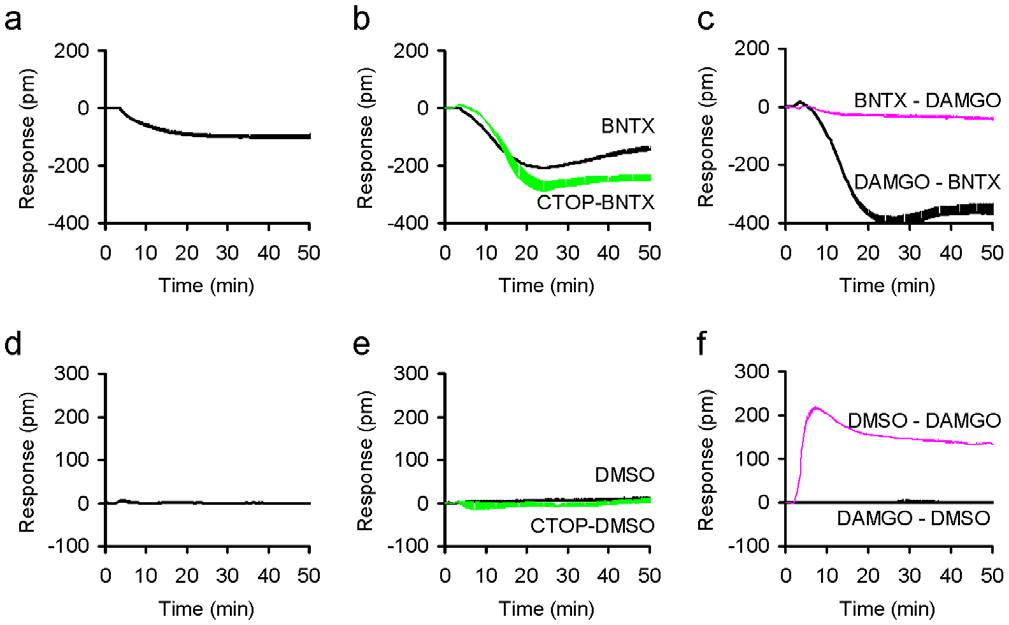
The DMR characteristics of BNTX and the negative control (0.1% DMSO). (a) The BNTX DMR in HEK-293 cells; (b) the BNTX DMR in the buffer treated (BNTX) and CTOP pretreated (CTOP – BNTX) HEK-MOR cells; and (c) the DAMGO DMR in the BNTX pretreated cells (BNTX – DAMGO) in comparison with the BNTX DMR in the DAMGO pretreated cells (DAMGO – BNTX); (d) the DMR induced by DMSO in HEK293; (e) the DMSO DMR in HEK-MOR or the CTOP pretreated HEK-MOR cells; (f) the DAMGO DMR after pretreatment with DMSO (DMSO – DAMGO) and the DMSO DMR after pretreatment with DAMGO (DAMGO –DMSO) in HEK-MOR cells. Each curve represents the average of duplicates.

The results of this analysis are shown in column groups A to F of the heat map (Fig. 4). Of all the compounds tested, the DOR-selective antagonist BNTX appeared to exhibit the most significant off-target activity in parental HEK293 cells – BNTX produced a negative DMR (N-DMR) in parental HEK 293 cells (Fig. 5a), suggesting that BNTX activated an endogenous cellular target. Further, BNTX also led to an N-DMR in HEK-MOR cells greater than that in the parental cell line (Fig. 5b), suggesting that the BNTX response is sensitive to the expression of the MOR. However, CTOP only slightly altered the BNTX-induced DMR response in HEK-MOR cells (Fig. 5b), confirming that the BNTX response is largely due to the activation of an endogenous target. In addition, BNTX pretreatment inhibited the DAMGO response in HEK-MOR cells (Fig. 5c), suggesting that BNTX is also an antagonist for the MOR. Finally, BNTX gave rise to an N-DMR in the DAMGO pretreated HEK-MOR cells that is greater than that in the untreated cells (Fig. 5c), suggesting that BNTX blocked the DAMGO-induced MOR activation. Taken together, these results suggest that BNTX has a previously unrecognized antagonistic effect against the MOR, and also interacts with an unknown endogenous target as described by others [36].

In control experiments, we found that the assay buffer containing 0.1% DMSO, a concentration identical to that in all ligand solutions, did not lead to any obvious DMR in parental HEK293 cells (Fig. 5d)**,** in HEK-MOR cells or in HEK-MOR cells with CTOP-pretreatment **(** Fig. 5e **)**. In addition, DMSO alone did not have an effect on DAMGO-mediated signaling whether DMSO was added before or after DAMGO stimulation (Fig. 5f).

### Antagonist supercluster

**Figure 6:**
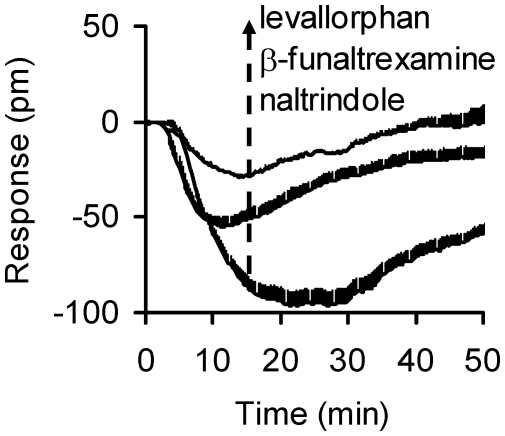
The DMR signals induced by levallorphan, β-funaltrexamine, and naltrindole in the DAMGO-activated HEK-MOR cells. Each curve represents the average of duplicates.

The predominant feature of the antagonist super cluster is that all ligands in this group, except for salvinorin A, produced a negative DMR (N-DMR) response in HEK-MOR cells after pretreatment with DAMGO (Column group D in Fig. 4). The full N-DMR response exhibited by three representative antagonists (levallorphan, β-funaltrexamine, and naltrindole) is shown in details in Fig. 6.

The antagonist supercluster can be further subdivided into three subclusters. The subcluster 1.1 consists of naltrexone, β-funaltrexamine, nalbuphine, levallorphan, and SKF10047. Naltrexone appeared to be a neutral antagonist lacking partial agonist activity at the MOR. The four remaining ligands produced a noticeable P-DMR in HEK-MOR cells, suggesting that the four ligands are partial agonists for the MOR. The partial agonism of these ligands was further confirmed by the fact that the CTOP pretreatment blocked their DMR response. The fact that pretreatment with all ligands in the subcluster completely blocked the DAMGO response suggests that these ligands are potent antagonists.

The antagonist subcluster 1.2 consists of naltrindole, naloxone methionone, naloxonazine, and naloxone HCl. All ligands in this subgroup were inactive in parental HEK293 cells, as well as in HEK-MOR cells, suggesting that the common feature of these ligands, when compared to subcluster 1.1, is the lack of partial agonist activity. Naloxone HCl and naloxonazine appeared to be more potent inhibitors of the DAMGO response than the other two ligands in this subcluster.

The antagonist subcluster 1.3 consists of salvinorin A, nor-binaltorphine, and N-benzylnaltrindole. The common feature of the antagonists in this subcluster is that all three ligands appeared to be less potent inhibitors of the DAMGO DMR response than those in subclusters 1.1 and 1.2. However, salvinorin A is distinct in that it appears to possess partial agonist activity at the MOR. Treatment of HEK-MOR cells with salvinorin A produced a noticeable P-DMR signal, while pretreatment of HEK-MOR cells with salvinorin A followed by exposure to CTOP resulted in an N-DMR response.

### Agonist supercluster

All ligands in the agonist supercluster produced a P-DMR in HEK-MOR cells, indicating that they all exhibit some degree of agonism at the MOR. When HEK-MOR cells were pretreated with either DAMGO or CTOP, addition of supercluster 2 agonists produced an attenuation of the DMR response compared to their respective DMR in the DMSO-treated HEK-MOR cells. When HEK-MOR cells were pretreated with ligands in this supercluster, followed by exposure to CTOP, CTOP produced an N-DMR. These results suggest that the ligands in the agonist supercluster are acting predominantly at the MOR, although it is possible that these ligands may also act allosterically or have some off-target activity.

The agonist supercluster can be further divided into three subclusters. The agonist subcluster 2.1 is composed of endomorphin-2, (met^5^)-enkephalin, dermorphin, DAMGO, (D-Ala^2^, D-Leu^5^) enkephalin, dynorphin A 1-8, dynorphin B, DSLET, (Leu^5^)-enkephalin, syndyphalin SD-25, endomorphin-1, TAPP, DAMME and ICI 199,441. Except for ICI 199,441, all ligands in this subcluster were inactive in parental HEK-293 cells, but exhibited agonist activity in the HEK-MOR cells. Pretreatment of HEK-MOR cells with ligands in this subcluster caused the MOR to become completely desensitized, since cells failed to respond to repeated stimulation with DAMGO. Similarly, when HEK-MOR cells were pretreated with DAMGO, they failed to respond to repeated stimulation with the ligands in this subcluster. The cross-desensitization between DAMGO and ligands in the subcluster 2.1 is consistent with the idea that ligands in this group act specifically at MOR sites and that they possess sufficient potency to desensitize MORs. Further, pretreatment of HEK-MOR cells with CTOP blocked the DMR response induced by these ligands, while CTOP produced an N-DMR response in HEK-MOR cells after pretreatment with any of these ligands. Together, these results support the view that ligands in this subcluster are potent agonists at MOR sites.

The agonist subcluster 2.2 consists of DIPPA, BRL-52537, BUBUC, dynorphin A 1-13, NNC 53-0532, DPDPE, DALDA, morphiceptin, (-)-norcodeine, and U-62066. Pretreatment of HEK-MOR cells with DAMGO fully inhibited the DMR response produced by the addition of ligands in this subcluster. In contrast with the ligands in subcluster 2.1, all ligands in this subcluster produced only a partial attenuation of the DAMGO-induced DMR response when the cells were pretreated with the subcluster 2.2 ligands. These results suggest that the ligands in subcluster 2.2 act at MOR sites, but are not as potent agonists as the ligands in subcluster 2.1. Notably, DIPPA and U-62066, previously classified as KOR-specific [37], [38], were slightly distinct from other ligands in this subcluster based on cluster analysis of all 13 assay results.

The agonist subcluster 2.3 consists of U-69593, tramadol, U-50488, deltorphin II and nociceptin 1-13. The members of this group are a mixture of KOR, DOR, MOR, and ORL-1 agonists. All ligands in this subcluster produced a P-DMR smaller than the DMR exhibited by agonists in the subcluster 2.1, suggesting that the ligands in this group act as partial or weak agonists at MOR sites. Pretreatment with DAMGO or CTOP completely blocked the cell response to ligands in this subcluster, supporting the view that subcluster 2.3 ligands do in fact act primarily at the MOR sites. In contrast, pretreatment of HEK-MOR cells with ligands in subcluster 2.3 did not fully desensitize MOR sites to subsequent treatment with DAMGO. These results suggest that the ligands in this subcluster are weak MOR agonists.

**Figure 7:**
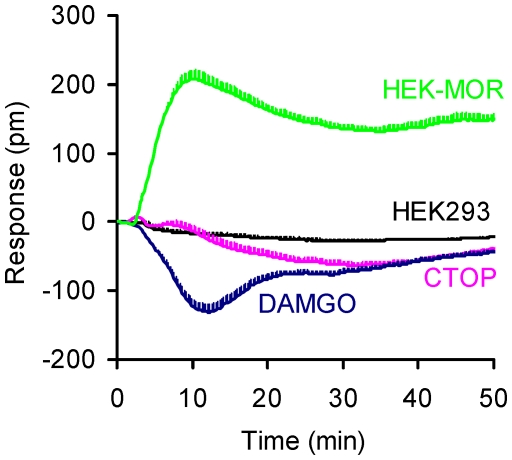
The ICI 199441-induced DMR in distinct molecule-treated cells. The cells were HEK-293 cells (HEK293), HEK-MOR (HEK-MOR), or CTOP- (CTOP), or DAMGO- (DAMGO) pretreated HEK-MOR cells. Each curve represents the average of duplicates.

All of the ligands tested, except for ICI 199,441, in the agonist supercluster were inactive in parental HEK-293 cells. ICI 199,441 produced a P-DMR in HEK-MOR cells, but a small N-DMR in the parental cell line and in CTOP- or DAMGO-pretreated cells (Fig. 7). Similar to BNTX, the ability of ICI 199,441 to produce an N-DMR response in multiple assays strongly suggests that it interacts with an unknown endogenous receptor.

### G-protein signaling

**Figure 8:**
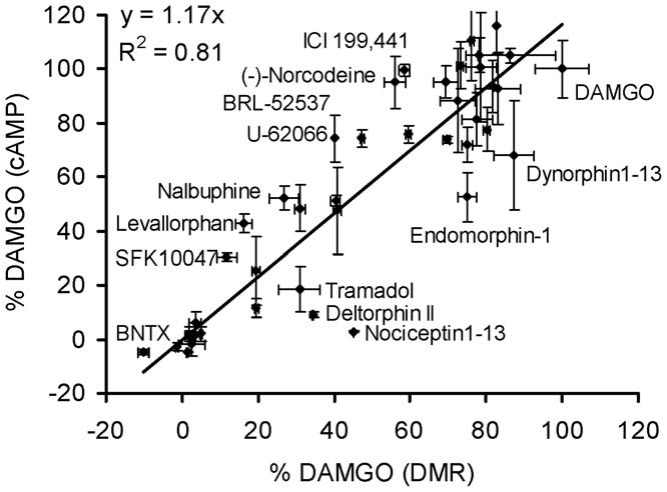
The comparison between the DMR responses and the whole cAMP responses induced by opioid ligands. Both were obtained in HEK-MOR cells. All data represent the mean ± s.d. of 2 independent measurements (n = 4).

To determine whether DMR assay is reflective of traditional assays that measure the biochemical response of cells to opioid agonist-induced MOR activation, we examined the relationship between the DMR response and whole cell cAMP changes produced by exposure of HEK-MOR cells to the library of opioid ligands. Results showed that there is a good linear correlation between cAMP and DMR assay results with an R^2^ of 0.81 (Fig. 8). The slope of 1.17 observed suggests that the whole cell cAMP signals are more easily saturated than the DMR signals, making it difficult it to resolve strong partial agonists from full agonists. Notably, a subset of ligands including nociceptin 1-13, deltorphin II, tramadol, endomorphin-1, and dynorphin A 1-13 were biased towards DMR, while others including SFK10047, levallorphan, nalbuphine, U-62066, BRL-52537, norcodeine, and ICI-199441 were biased towards cAMP. Nonetheless, these results support the validity of DMR assay when compared to conventional GPCR cell-based assays.

It is well established that MOR signaling is primarily coupled to the G_αi_ class of heterotrimeric G-proteins [39]. However, recent work has challenged this dogma, and suggests that in addition to G_αi_, the MOR may also signal through other G-protein subtypes [40]. It has been shown that the DMR assay is capable of recognizing all G-protein coupled pathways [20], [22]. In order to deconvolute the signaling pathways, we analyzed the effect of G-protein inhibitors on MOR-mediated signaling induced by opioid ligands.

**Figure 9:**
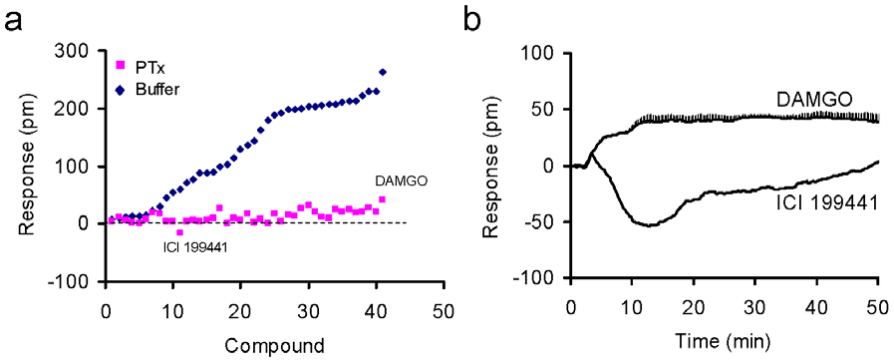
The sensitivity of opioid ligand-induced DMR to the pertussis toxin (PTx) pretreatment. (a) the comparison between the DMR induced by each ligand in the untreated and PTx-pretreated HEK-MOR cells, in which the maximal amplitudes were plotted.; (b) The DMR arising from ICI 199441 and DAMGO in the PTx-pretreated HEK-MOR cells. All data represent the mean ± s.d. of 2 independent measurements (n = 4).

First, we used pertussis toxin (PTx) to inhibit G_αi_ via ADP ribosylation of a Cys residue and uncoupling of the G protein from the receptor. Results showed that masking of G_αi_ by PTx inhibited the DMR response induced by all opioid agonists tested, except for DAMGO and ICI 199,441 (Fig. 9a). ICI 199,441 produced a small N-DMR in the PTx-treated cells, while DAMGO still led to a small P-DMR (Fig. 9b). These results suggest that virtually all of the MOR activity induced by the library ligands is G_αi_-coupled. However, DAMGO may also contain a G_αi_ -independent signaling component, while ICI 199,441 is likely to activate an endogenous target and/or may also mediate G_αi_-independent signaling.

**Figure 10:**
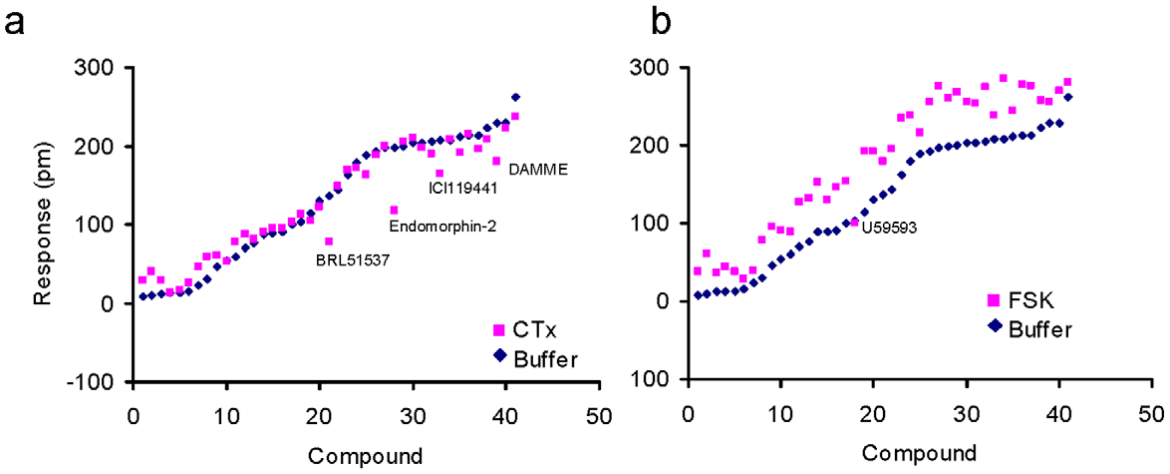
The sensitivity of opioid ligand induced DMR to the pretreatment with forskolin (FSK) and cholera toxin (CTx). The maximal DMR amplitudes of all opioid ligands were compared in HEK-MOR after pretreatment with: (a) buffer versus CTx; and (b) buffer versus forskolin.

Next, we used cholera toxin (CTx) to activate G_αs_ proteins by ADP ribosylation of an Arg residue of the protein. CTx treatment is known to lead to cAMP production [41]. CTx treatment had little effect on the DMR response induced by most of the agonists tested, except for DAMME, ICI 199,441, endomorphin-2 and BRL-51537 (Fig. 10a). CTx was found to reduce the DMR response induced by DAMME, ICI 199,441, endomorphin-2 or BRL-51537, suggesting that these four ligands may also signal through a G_αs_ component. Interestingly, except for naltroxone and naltrindole, all ligands in the antagonist cluster generally produced more pronounced DMR responses in CTx-pretreated cells than in untreated cells. This suggests that the increased basal G_αs_ activity after CTx pretreatment permitted these antagonists to act as partial agonists.

Lastly, we used forskolin to increase the basal cAMP level before stimulation with opioid ligands. Forskolin is an adenylate cyclase activator and is widely used to increase intracellular cAMP levels [42], thus leading to the potentiation of G_αi_-coupled signaling via heterologous sensitization [43]. We found that, with the exception of U69593, pretreatment of HEK-MOR cells with forskolin up-regulated the DMR response normally produced by opioid ligands (Fig. 10b). Together, these results suggest that the opioid agonist-induced DMR signals are predominantly the result of G_αi_-mediated signaling.

### Kinase Pathways

We used a panel of specific kinase inhibitors to determine the potential pathway biased activity of opioid ligands [44]. We chose U0126 to inhibit the ERK1/2 pathway [45], SB202190 to inhibit the p38 pathway [46], SP600125 to block JNK-mediated signaling [47] and LY294002 to inhibit signaling through PI3K [48]. Here, HEK-MOR cells were first pretreated with one of the kinase inhibitors, followed by exposure to an opioid ligand. For each ligand we compared the maximal DMR response within 15 min post-stimulation, and the response at 30 min post-stimulation.

**Figure 11:**
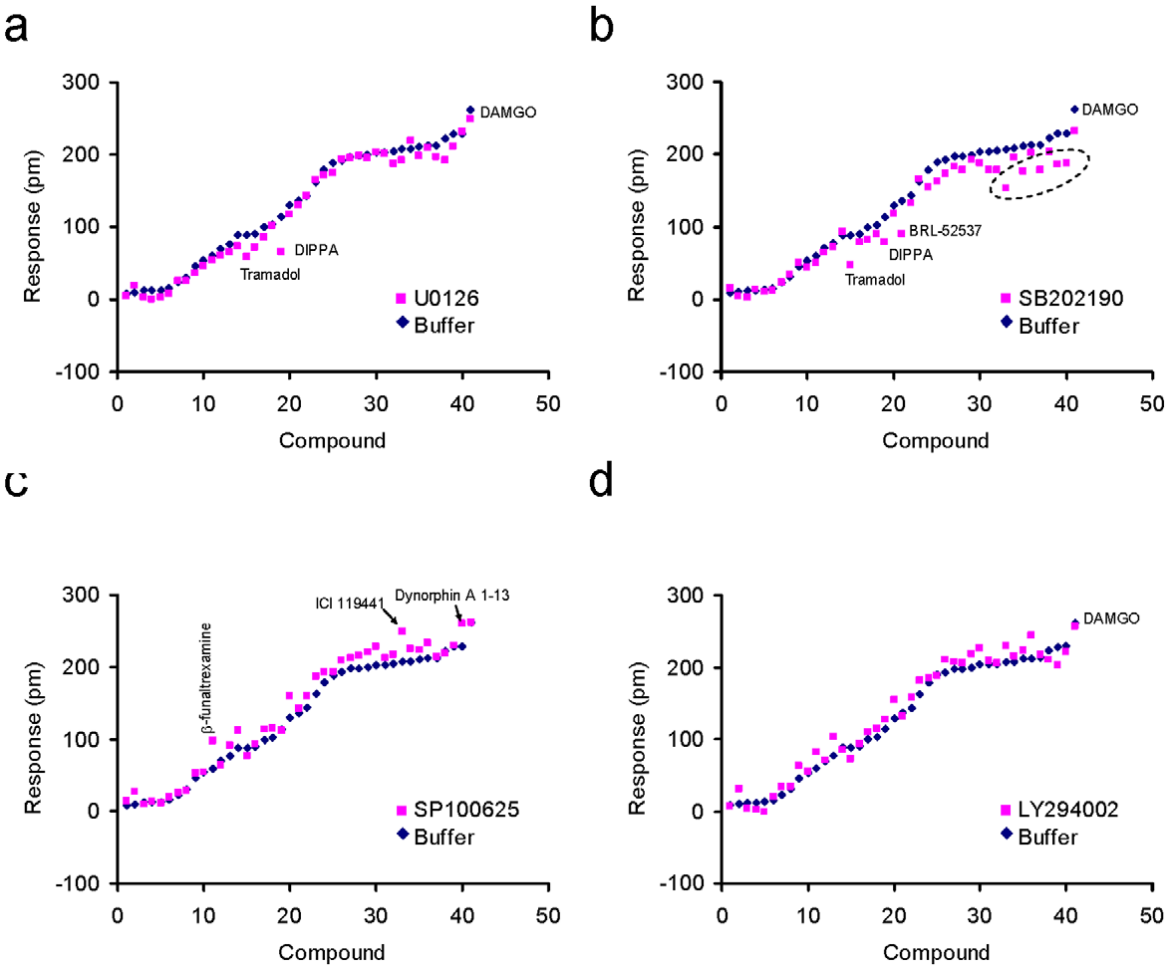
The comparison of the maximum responses of opioid ligands between the buffer- and inhibitor-pretreated HEK-MOR cells. (a) U0126; (b) SB202190; (c) SP100625 and (d) LY294002. The broken circle referred to ICI199441, Leu^5^-enkephalin, DSLET, DAMME, and dynorphin A 1-13 from left to right, respectively.

First, we compared the maximal response for each ligand-induced DMR response in HEK-MOR cells with that in HEK-MOR cells after pretreatment with each of the kinase inhibitors. As shown in Fig. 11, the maximal DMR response for each ligand in cells pretreated with kinase inhibitors was, in general, comparable to that in untreated cells. However, the DMR signals induced by tramadol and DIPPA were sensitive to pretreatment with U0126 (Fig. 11a). Further, DMR signals produced by a subset of agonists including DIPPA, tramadol, BRL-52537, ICI 199,441, Leu^5^-enkephalin, DSLET, DAMME, and dynorphin A 1-13 were significantly reduced in cells pretreated with the p38 MAPK inhibitor SB202190 compared to controls (Fig. 11b). The DMR response induced by β-funaltrexamine, ICI 199,441 and dynorphin A 1-13 was enhanced by SP100625 pretreatment (Fig. 11c). LY294002 pretreatment appeared to have little, if any, effect the maximal DMR response induced by any of the ligands (Fig. 11d).

**Figure 12:**
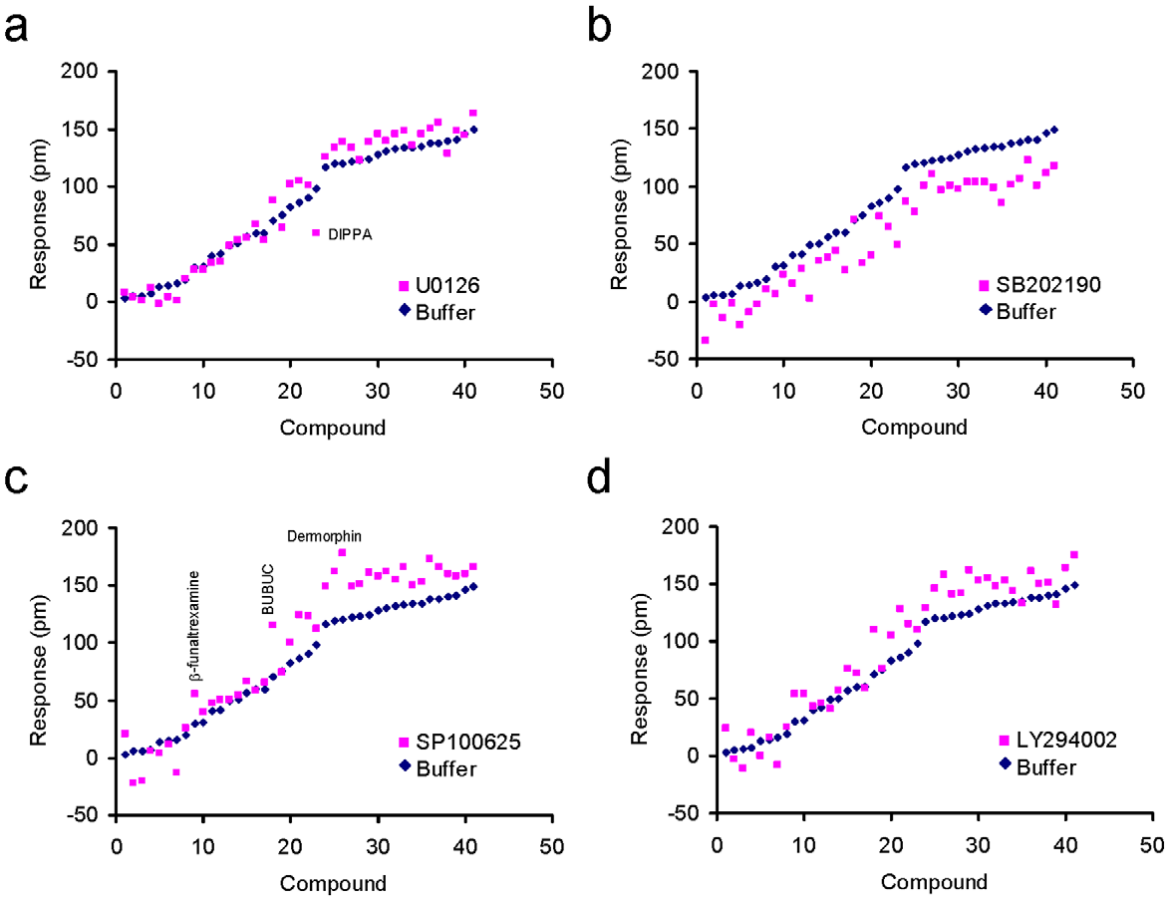
The comparison of the real responses (30 min post-stimulation) of opioid ligands between the buffer- and inhibitor-pretreated HEK-MOR cells. (a) U0126; (b) SB202190; (c) SP100625 and (d) LY294002.

We next compared the late response (*i.e.*, the DMR amplitude at 30 min post-stimulation) of each ligand-induced DMR in HEK-MOR cells with that in HEK-MOR cells after pretreatment with kinase inhibitors. The results indicate distinct sensitivity of the late responses for many ligands to the pretreatment with distinct kinase inhibitors. The late response induced by DIPPA was significantly suppressed by U0126 pretreatment (Fig. 12a), while the late responses induced by most agonists were significantly suppressed by SB202190 pretreatment (Fig. 12b). However, SP100625, in general, selectively potentiated the DMR induced by strong partial and full agonists (Fig. 12c). SP100625 also potentiated both BUBUC- and β-funaltrexamine-induced DMR. LY294002 impacted the late responses induced by most ligands, but no clear trend was observed (Fig. 12d). These results suggest that MOR ligands exhibit functional selectivity in their ability to trigger pathway biased agonism.

## Discussion

We have used a label-free integrative pharmacology on-target approach to characterize a library of opioid ligands at the MOR. These studies were facilitated by the HEK-MOR cell line, since this cell line stably expresses the MOR and is devoid of kappa or delta receptors. We have also taken advantage of the ability of label-free Epic® system to monitor the DMR responses with high throughput. This system has allowed us to perform multiple assay formats for studying the activity of the library of opioid ligands tested, and obtain an integrated picture of their functional selectivity.

This approach differs significantly from traditional pharmacological methods in several important aspects. First, the DMR assay utilizes a whole cell readout to probe the activity of individual receptors as well as the integrated cellular response to drugs [18]. Second, DMR assays are performed in real time on live cells and do not require radioactive labeling or other types of manipulations that might skew or influence the behavior of cells in response to agonist or antagonist drugs. Further, the combinations of different assay formats enabled by DMR allow one to distinguish between various ligands based on their specificity, relative potency and mechanisms of action at specific GPCR sites. DMR assays are highly sensitive, thus making it possible to examine the ligand-directed functional selectivity at the MOR.

The results obtained using DMR assays are, in general, consistent with those obtained using classical radioligand-based approaches (Table S1). Compounds previously identified as MOR agonists exhibited MOR selectivity and activated G_αi_ signaling pathways as measured by DMR. Compounds previously identified as antagonists behaved accordingly, and were found to inhibit the DMR response induced by known MOR agonists. These studies provide compelling support for the idea that the DMR assay can be utilized to analyze the effect of opioid ligands on the MOR.

The DMR assays are sensitive, and have allowed us to uncover several unexpected and atypical characteristics of known opioid ligands. First, of all the compounds tested, BNTX gave the most anomalous DMR response. BNTX has previously been classified as a selective DOR antagonist. Cluster analysis further suggests that BNTX is distinct from all other ligands tested (Fig. 4). The DMR profiling of BNTX suggests that beside its antagonist activity at the MOR, BNTX also produced a negative DMR response in parental HEK-293 cells and an even greater negative DMR in HEK-MOR cells, suggesting that BNTX interacts with another target endogenously expressed in HEK293 cells. The unique DMR profiles of BNTX highlight the power of the DMR assay to identify the pharmacological activity of any non-selective ligands that act on both endogenous targets and the engineered receptors. Second, DMR profiling suggests that ICI 199,441 is not only an agonist for the MOR, but also activates another endogenous target. ICI 199,441 was previously classified as a full agonist for KOR. Third, for several DOR-specific ligands, DSLET was the only putative delta agonist that produced a DMR response similar to those induced by full MOR agonists in HEK-MOR cells. DMR profiling, confirmed by cAMP assay results, suggests that DSLET is a full agonist for the MOR. Fourth, nalbuphine, β-funaltrexamine and levallorphan were found to be partial agonists for the MOR. Nalbuphine and β-funaltrexamine are often considered to be MOR-specific antagonists, while levallorphan is classified as a pan-opioid receptor antagonist [49]–[51]. Taken together, label-free on-target pharmacology profiling has revealed that several of the ligands in the library exhibit novel activities that are undetectable using traditional pharmacological techniques.

We have utilized DMR to examine the functional selectivity of opioid ligands at the MOR. We first used DMR assays to examine the functional selectivity of the ligands for coupling to heterotrimeric G-proteins. Our results are consistent with the view that all MOR agonists examined signal predominantly through G_αi_. However, blocking G_αi_ with PTx revealed that both DAMGO and ICI 199,441 also signal through a G_αi_ -independent signaling component. Permanent activation of G_αs_ with CTx showed that DAMME, ICI 199,441, endomorphin-2 or BRL-52537 may also couple MOR signaling through a G_αs_ component. These results suggest that specific opioid ligands may confer functional selectivity by inducing the MOR to couple to alternate subsets of heterotrimeric G-proteins.

Opioid receptor activation leads to the downstream activation of numerous kinase pathways. These pathways are differentially activated by various opioid ligands [4], [51]. It has been proposed that the specification of kinase signaling pathways is crucial to understanding the underlying physiology of addiction, but little is currently known about the functional selectivity of specific opioid ligands to activate distinct downstream signaling pathways [40]. We believe that the application of DMR technology to MOR signaling will help bridge this gap in knowledge by allowing us to understand the intricate network of modulations among opioid ligands, receptors, and downstream protein kinase signaling pathways.

DMR assays showed that specific opioid ligands appear to exhibit functional selectivity towards distinct kinase signaling pathways. For example, the kinase inhibitor SP100625 appeared to potentiate the DMR signal induced by β-funaltrexamine, ICI 199,441 and dynorphin A 1-13, suggesting that these ligands selectively activate the JNK kinase signaling pathway. However, since these ligands also gave a small N-DMR in parental HEK-293 cells, it is possible that these ligands can activate the JNK signaling pathway via a non-MOR receptor signaling pathway. Interestingly, DIPPA, tramadol, BRL-52537, ICI 199,441, Leu^5^-enkephalin, DSLET, DAMME, and dynorphin A 1-13 all produced a DMR sensitive to the p38 MAPK inhibitor SB202190, suggesting that these ligands are biased towards the p38 MAPK pathway. DAMGO has previously been implicated as signaling through this pathway using conventional molecular assays [52]. However, we found that the DAMGO-induced DMR response was not completely blunted by the p38 inhibitor SB202190 using whole cell DMR assays. Nonetheless, this is the first report implicating DIPPA, tramadol and BRL-52537 in activation of the p38 kinase signaling pathway. Our results suggest that the p38 pathway may play a more vital role in MOR-mediated signaling than has been previously appreciated [53].

The biochemical and pharmacological characterization of opioid receptors has proven to be a difficult problem. First, native cells often express more than one opioid receptor [54], and yet, opioid ligands often exhibit relatively poor selectivity for different opioid receptors [55]. Second, opioid receptors, like other GPCRs, are believed to form homo- or heterodimers, which often results in signaling distinct from monomeric receptors [9], [56]–[61]. The dual potency in DMR exhibited by fentanyl and endomorphin-1 is indicative of the potential existence of MOR homodimers [62], [63]. Similar results have previously been reported for endomorphin-1 [63]. Third, the plasticity of receptor conformations [6], [7] implies that distinct ligands could result in pathway-selective activity via stabilization of a specific receptor conformation or a specific set of conformations [64]–[66]. Fourth, differences in intracellular environments may also affect ligand-mediated pharmacology, given that biological functions are often cell context dependent. Fifth, the MOR, like many GPCRs, has splice variants which often occur within the domains of the receptor involving interactions with other intracellular proteins, thus resulting in distinguishable functional consequences including altered receptor trafficking [67], [68]. High sensitivity, coupled with broad pathway coverage, makes the non-invasive, manipulation-free, and label-free biosensor cell-based assay an attractive means to elucidate the signaling of opioid receptors, and by extension, all GPCRs and their splice variants. Therefore, the iPOT approach represents a novel and practical means to study receptor-based pharmacology in multiple dimensions, and provides a global view of ligand pharmacology which should accelerate the drug discovery process and help prioritize MOR receptor active compounds for *in vivo* testing.

## Methods

### Materials and reagents

Pertussis toxin, cholera toxin, forskolin and dimethyl sulfoxide (DMSO) were purchased from Sigma-Aldrich (St. Louis, MO). DAMGO, DPDPE, BRL-53527, CTOP, naltrindole hydrochloride, norbinaltorphimine, U0126, SB202190, SP600125, and LY294002 were purchased from Tocris Biosciences (Ellisville, MO). The Opioid Compound Library (consisting of 64 compounds of pan-specific and receptor subtype-specific agonists and antagonists, each at 10 mM in DMSO) was obtained from Enzo Life Sciences (Plymouth Meeting, PA). All tissue culture media and reagents were purchased from Invitrogen (Calrsbad, CA). Fibronectin-coated Epic® biosensor microplates and polypropylene compound source plates were obtained from Corning Inc (Corning, NY).

### Cell Culture

HEK293 cells were obtained from American Type Tissue Culture (Manassas, VA) and cultured in Dulbecco's modified Eagle's medium (DMEM GlutaMAX-I) supplemented with 10% non-heated inactivated fetal bovine serum, 100 units/ml penicillin, and 100 g/ml streptomycin. The HEK-MOR cell line was a generous gift from Dr. Mark von Zastrow (University of California, San Francisco). HEK-MOR cells express FLAG-tagged wild type human mu opioid receptor (MOR1) with a B_max_ of 2.5 pmoles/mg cell protein [60]. These cells were grown in complete DMEM GlutaMAX-I containing 400ug/ml geneticin.

### Dynamic mass redistribution (DMR) assays

Whole cell DMR assays were performed using the Corning Epic® system as previously described [22]–[25], [69]. One day prior to performing DMR assay, cells were seeded onto fibronectin-coated Epic® microplates at a density of 16,000 cells/40 µL/well for HEK293 cells and 20,000 cells/40 µL/well for HEK-MOR cells. After seeding, the Epic® microplates were incubated for 30 min at room temperature, and then transferred to a humidified incubator (37°C, 5% CO_2_) for 20–24 hrs.

Prior to initiating the DMR assay, cells were washed with assay buffer (Hank's balanced salt solution with 20 mM HEPES) and transferred to the Epic® reader for 1 hr at 26°C. DMR was monitored before and after addition of compounds. In a one step assay, a library ligand was added directly to cells and the DMR was monitored for 1 hr. In a two step assay, HEK-MOR cells were preconditioned with a series of probe molecules to achieve a wide range of chemical environments, which, in turn, manifest the specificity, relative potency and efficacy, and modes of action of the drugs. Specifically, cells were pretreated with either 0.1% DMSO (the positive control), 10 µM CTOP, 10 µM DAMGO, 10 µM opioid ligand, 100 ng/ml PTx, 400 ng/ml CTx, 10 µM forskolin, 10 µM U0126, 10 µM SB202190, 10 µM SP100625, or 10 µM LY294002 for the times indicated (Table 1). Cells were then stimulated with an opioid ligand (DAMGO, CTOP, or the library compounds), whose responses were recorded in real time and used for similarity and correlation analysis. We screened a library of 64 opioid ligands. Twenty-two ligands that gave a K_i_ value for the MOR greater than 5 µM were not included in the analysis. The remaining 42 library ligands were analyzed using the DMR assay.

### Whole cell cAMP assays

Inhibition of the forskolin-stimulated cAMP accumulation in HEK293 and HEK-MOR cells was performed using the cAMP-GLO assay kit (Promega, Madison, WI), according to the manufacturer's instructions. Briefly, cells were plated in 384-well poly-lysine-coated tissue culture plates (BD Bioscience, Cat# 354660) at a seeding density of 15,000 cells per well. After 24 hrs incubation at 37°C, media was removed, and cells were incubated with 10 µM of library compounds in the presence of 5 µM forskolin in cAMP induction buffer for 30 minutes. The reaction was terminated by adding lysis buffer and luminescence was measured using Tecan SafireII reader.

### Data visualization and clustering

For each opioid ligand, thirteen DMR assays were performed that measured receptor specificity, G-protein coupling, and downstream kinase pathway selectivity. For each DMR assay, cellular responses were monitored every 15 seconds over a period of 50 minutes. Data was then analyzed at three distinct time points (3 min, 9 min, and 30 min) after ligand addition. Each data point represents the average of two replicates. For visualization, the real-time responses were color coded to illustrate relative differences in DMR signal strength. The red color refers to a positive value, the black a value near zero, and the green color represents a negative value. Differences in color intensity illustrate differences in signal strength. In the ligand matrix, each column represents one DMR response at a particular time under a specific assay condition, and each row represents one ligand. Every row and column carries equal weight. The Ward hierarchical clustering algorithm and Euclidean distance metrics [34] were used for generating heat maps and clustering the DMR profiles. To assist with direct visualization of DMR characteristics of each ligand in an assay, we did not carry out similarity analysis among distinct columns. The 13 assays were designated A to M (Fig. 4), and each assay was arranged in three consecutive columns to form a column group.

### Statistical analysis

For profiling, two independent measurements, each done in duplicate, were performed. In order to be included in the analysis, all replicates passed the 2 sigma coefficient of variation test. Drugs whose DMR responses failed the statistical test were re-screened. At least two replicates were included for the final analysis. For dose responses, at least two independent measurements, each done at least in duplicate, were performed to calculate the mean responses and the standard deviations (s.d.).

## Supporting Information

Table S1 Opioid ligands, their binding affinity, and references.
(PDF)
