# Supplementary material for: Ligand-Directed Functional Selectivity at the Mu Opioid Receptor Revealed by Label-Free Integrative Pharmacology On-Target
Source: PLoS One. 2011 Oct 7;6(10):e25643. doi: 10.1371/journal.pone.0025643 (PMC3189208; doi:10.1371/journal.pone.0025643)
Supplement: Table S1 — Opioid ligands, their binding affinity, and references. (PDF) [file pone.0025643.s001.pdf]

Supplementary Information

Ligand-directed functional selectivity at the mu opioid receptor revealed by label-free integrative pharmacology on-target

Megan Morse[1], Elizabeth Tran[2], Haiyan Sun[2], Robert Levenson[1,*] and Ye Fang[2,*]

[1.] *Department of Pharmacology, Pennsylvania State University College of Medicine, Hershey, PA 17033 United States*

[2.] *Biochemical Technologies, Science and Technology Division, Corning Inc., Corning, NY 14830, United States*

Supplementary Table 1 Opioid ligands, their binding affinity, and references

Table S1 Opioid ligands, their binding affinity, and references

Compound	Pharmacology	Ki (nM)	Kd (nM)	EC50 (nM)	IC50 (nM)	Ref
Naloxonazine	Opioid antagonist	0.054				1
CTOP	Mu Antagonist		0.18			1
Syndyphalin SD-25	Mu agonist				0.29	2
DAMME	Mu agonist				0.33	3
Dermorphin	Mu agonist		0.33			4
beta-Funaltrexamine	Mu antagonist	0.33				1
Endomorphin-2	Mu agonist		0.43			5
TAPP	Mu ligand		0.47			6
Endomorphin-1	Mu agonist	0.67				5
Naltrexone	Opioid antagonist	0.77				7
DAMGO	Mu agonist		1.1			9
DALDA	Mu agonist		1.69			9
Levallorphan	Opioid antagonist	1.7				7
nor-Binaltorphimine	Kappa antagonist	2.2				1
Naloxone HCl	Opioid antagonist	2.4				10
(Met5)-Enkephalin	Mu agonist	3				11
Deltorphin II	Delta agonist		3.3			4
(Leu5)-Enkephalin	Mu agonist		7.4			11
Nalbuphine	Mu antagonist/kappa agonist	11				1
N-Benzylnaltrindole	Delta antagonist				11.2	12
Dynorphin B	Kappa agonist	13				13
BNTX	Delta antagonist	18				4
Morphiceptin	Opioid agonist				19.8	14
Dynorphin A (1-8)	Kappa agonist	22.3				13
Naloxone methiodide	Opioid antagonist	28.9				15
Dynorphin A (1-13)	Kappa agonist		31			16
Naltrindole	Opioid antagonist	64				4
Naltriben	Delta antagonist.		80.8			1
DSLET	Delta agonist		120			17
NNC 63-0532	ORL1 agonist	140				10
DADLE	Opioid agonist		150			16
U-62066	Kappa agonist	252				18
(-)-Norcodeine	Opioid agonist		266.9			19
Nociceptin (1-13)	ORL1 agonist	270				20
DPDPE	Delta Agonist		600			8
(-)-U-50488	Kappa partial agonist		830			16
BRL52537	Kappa agonist	1560				21
U-69593	Kappa agonist	1600				10
Salvinorin A	Kappa agonist			1728		22
DIPPA	Kappa antagonist				1799	23
SKF10047	Opioid agonist/antagonist	1900			16	
Tramadol	Mu agonist	2400				24
ICI 199,441	Kappa agonist		4500			25

References

1. Raynor K, Kong H, Yasuda K, Yu L, Bell GI, et al. (1994) Pharmacological characterization of the cloned kappa-, delta-, and mu-opioid receptors. Mol Pharmacol 45: 330-334.
2. Quirion R, Kiso Y, Pert CB (1982) Syndyphalin SD-25: a highly selective ligand for mu opiate receptors. FEBS Lett 141: 203-206.
3. Leslie FM, Tso A, Hurlbut DE (1982) Differential appearance of opiate receptor subtypes in neonatal rat brain. Life Sci 31(12-13):1393-1396.
4. Reisine T (1995) Opiate receptors. Neuropharm 34: 463-472.
5. Goldberg IE, Rossi GC, Letchworth SR, Mathis JP, Ryan-Moro J, et al. (1998) Pharmacological characterization of endomorphin-1 and endomorphin-2 in mouse brain. J Pharmacol Exp Ther 286:1007-1013.
6. Spetea M, Otvos F, Toth G, Nguyen TMD, Schiller PW, et al (1998) Interaction of agonist peptide [3H]Tyr- -Ala-Phe-Phe-NH_2 with μ-opioid receptor in rat brain and CHO-μ/1 cell line. Peptides 19: 1091-1098.
7. Carroll JA, Shaw JS, Wickenden AD (1988) The physiological relevance of low agonist affinity binding at opioid μ-receptors. Br J Pharmacol 94: 625-631.
8. Amiche M, Sagan S, Mor A, Delfour A, Nicholas P (1989) Dermenkephalin (Tyr-D-Met-Phe-His-Leu-Met-Asp-NH_2): A potent and fully specific agonist for the delta opioid receptor. Mol Pharmacol 35: 774-779.
9. Schiller PW, Nguyen TM, Chung NN, Lemieus C (1989) Dermorphin analogs carrying an increased positive net charge in their "message" domain display extremely high mu-opioid receptor selectivity. J Med Chem 32: 698-703.
10. Titeler M, Lyon RA, Kuhar MJ, Frost JF, Dannais RF, et al (1989) μ Opiate receptors are selectively labelled by [3H]carfentanil in human and rat brain. Eur J Pharmacol 167: 221-228.
11. Childers SR, Creese I, Chowman AM, Snyder SH (1979) Opiate receptor binding affected differentially by opiates and opioid peptides. Eur J Pharmacol 55: 11-18.
12. Korlipara VL, Takemori AE, Portochese PS (1994) N-Benzylnaltrindoles as long acting δ-opioid receptor antagonists. J Med Chem 37: 1882-1885.

13. Merg F, Filliol D, Usynin I, Bazv I, Bark N, et al (2006) Big dynorphin as a putative endogenous ligand for the κ-opioid receptor. J Neurochem 97: 292-301.
14. Fichna J, Do-Rego JC, Janecki T, Staniszewska R, Poels J, et al (2008) Novel highly potent μ-opioid receptor antagonist based on endomorphin-2 structure. Bioorg Med Chem Lett 18:1350-1353.
15. Lewanowitsch T, Irvine RJ (2003) Naloxone and its quaternary derivative, naloxone methiodide, have differing affinities for μ, δ, and κ opioid receptors in mouse brain homogenates. Brain Res 964: 302-305.
16. Tam SW (1985) (+)-[3H]SKF 10,047, (+)-[3H]ethylketocyclazocine, μ, κ, and δ phencyclidine binding sites in guinea pig brain membranes. Eur J Pharmacol 109: 33-41.
17. Creese I, Feinberg AP, Snyder SH (1976) Butyrophenone influences on the opiate receptor. Eur J Pharmacol 36: 231-235.
18. Wadenberg MLG (2003) A review of the properties of spiradoline: a potent and selective κ-opioid receptor agonist. CNS Drug Rev 9: 187-198.
19. Lotsch J, Skarke C, Schmidt H, Rohrbacher M, Hofmann U, et al (2006) Evidence for morphine-independent central nervous opioid effects after administration of codeine: Contribution of other codeine metabolites. Pharmacodynamics Drug Action 79: 35-48.
20. Halab L, Becker JA, Darula Z, Tourwe D, Kieffer BL, et al (2002) Probing opioid receptor interactions with azacycloalkane amino acids. Synthesis of a potent and selective ORL1 antagonist. J Med Chem 45: 5353-5357.
21. Chen TY, Goyagi T, Toung TJ, Kirsch JR, Hurn PD, et al (2004) Prolonged opportunity for ischemic neuroprotection with selective kappa-opioid receptor agonist in rats. Stroke 35: 1180-1185.
22. Rothman RB, Murphy DL, Xu H, Godin JA, Dersch CM, et al (2007) Salvinorin A: allosteric interactions at the μ-opioid receptor. J Pharmacol Exp Ther 320: 801-810.
23. Chang AC, Takemori AE, Portoghese PS (1994) 2-(3,4-Dichlorophenyl)-N-methyl-N-[(1S)-1-(3-isothiocyanatophenyl)-2-(1- pyrrolidinyl)ethyl]acetamide: an opioid receptor affinity label that produces selective and long-lasting kappa antagonism in Mice. J Med Chem 37: 1547–1549.

24. Gillen C, Haurand M, Kobelt DJ, Wnendt (2000) Affinity, potency and efficacy of tramadol and its metabolites at the cloned human μ-opioid receptor. Naunyn-schmiedeberg's Arch Pharmacol 362:116-121.
25. Costello GF, Main BG, Barlow JJ, Carrol JA, Shaw JS (1988) A novel series of potent and selective agonists at the opioid kappa-receptor. Eur J Pharmacol 151: 475-478.
